# Modeling population‐wide testing of SARS‐CoV‐2 for containing COVID‐19 pandemic in Okinawa, Japan

**DOI:** 10.1002/jgf2.439

**Published:** 2021-05-05

**Authors:** Kazuki Shimizu, Toshikazu Kuniya, Yasuharu Tokuda

**Affiliations:** ^1^ Department of Health Policy London School of Economics and Political Science London UK; ^2^ Faculty of Public Health and Policy London School of Hygiene and Tropical Medicine London UK; ^3^ Graduate School of System Informatics Kobe University Kobe Japan; ^4^ Muribushi Okinawa Center for Teaching Hospitals Okinawa Japan

**Keywords:** asymptomatic: health security, containment, COVID‐19, mass testing, public policy

## Abstract

To break the chains of SARS‐CoV‐2 transmission and contain the coronavirus disease 2019 (COVID‐19) pandemic, population‐wide testing has been practiced in various countries. However, scant research has addressed this topic in Japan. In this modeling exercise, we extracted the number of daily reported cases of COVID‐19 in Okinawa from October 1 to November 30, 2020, and explored possible scenarios for decreasing COVID‐19 incidence by combining population‐wide screening and/or social distancing policy. We reveal that permanent lockdown can be theoretically replaced by mass testing but sufficient target population at an adequate frequency must be mobilized. In addition, solely imposing a circuit breaker will not bring a favorable outcome in the long run, and mass testing presents implications for minimizing a period of lockdown. Our results highlight the importance of incentivizing citizens to join the frequent testing and ensure their appropriate isolation. This study also suggests that early containment of COVID‐19 will be feasible in prefectures where the mobility is low and/or can be easily controlled for its geographic characteristics. Rigorous investment in public health will be manifestly vital to contain COVID‐19.

## Graphical abstract

Population‐wide testing plays a vital role in containing COVID‐19, and possible scenarios for decreasing COVID‐19 incidence by combining population‐wide screening and/or social distancing policy are explored. We reveal that mass testing that mobilizes sufficient target population at an adequate frequency will help contain the pandemic, and solely imposing a circuit breaker will not bring a favorable outcome in the long run. Also, mass testing presents implications for minimizing a period of lockdown.

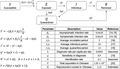

## 1 INTRODUCTION

To combat against the surge of coronavirus disease 2019 (COVID‐19) cases and break chains of SARS‐CoV‐2 transmission, many countries imposed various public health and social measures (PHSMs) in 2020. Especially, an unprecedented lockdown of cities, regions, and countries highly contributed to suppressing the virus.^1^ Although the economic loss and social burden on citizens, especially vulnerable populations, were critically featured, there has been a dispute on how to leverage socioeconomic activities with infection control measures in many countries where suppression and/or mitigation strategies were employed. It has been clarified that economic activities were subdued by social distancing policy, even at a voluntary basis.^2^ On the contrary, some countries or regions in the Western Pacific Region, such as China, Taiwan, Vietnam, or New Zealand, advanced for aggressively suppressing or eliminating COVID‐19, and have started to boost their socioeconomic activities.^3^ Australia has reflected on lessons learned in the winter season and advanced for aggressive suppression.

To contain the COVID‐19 pandemic, symptom‐based strategy, namely testing solely for symptomatic patients under the scheme of clinical medicine, is not feasible due to its large proportion of asymptomatic infections,^4^ and many countries rapidly ramped up the testing capacity, expanded testing targets, and arranged logistics for rigorous contact tracing and isolation. There has been an argument that weekly testing for the entire population, followed by contact tracing and quarantine, can break chains of transmission within a few weeks,^5^ and population‐wide testing has been practiced in various ways.^6^ China utilized a mass testing approach for early containment,^7^, ^8^ and some countries in Europe launched a novel approach. The Slovak Republic spearheaded for screening over 3.6 million citizens, and mass testing with other nonpharmaceutical interventions (NPIs) successfully reduced the prevalence of SARS‐CoV‐2.^9^ The United Kingdom firstly kicked off the trial of mass testing in Liverpool by a lateral flow test. In this scheme, positive cases were followed by confirmatory reverse transcription polymerase chain reaction (RT‐PCR) testing.^10^ Other countries, such as South Tyrol/Alto Adige in Italy, Austria and Slovenia, have already conducted or considered similar approaches.

While many Asian‐Pacific countries have rigorously contained the COVID‐19, Japan has employed suppression strategy. While maintaining the effective reproduction number at least below 1 was the core in this strategy, the government did not intensify PHSMs by its evolution and invest in ramping up testing for diagnosis, surveillance, and screening.^11^ Additional countermeasures and social support for high‐risk environments were not strengthened,^12^ and failure in containment brought the spread of the SARS‐CoV‐2 virus to the entire nation.^13^

After lifting the nationwide state of emergency in early May, Okinawa prefecture, whose population amounts to 1.46 million with 148 remote islands, aggressively suppressed the virus, and the number of COVID‐19 cases was maintained almost zero in May‐June 2020; however, extensive testing for stamping out the virus was not implemented. This, along with the importation from other prefectures and COVID‐19 outbreak in US military bases, brought a resurgence in late July, and Okinawa necessitated local state of emergency in August. After the summer 2020, the grand strategy became obscure, which consequently made Okinawa to face an increase in COVID‐19, and investigating a clear exit strategy has been an urgent research topic for protecting lives and livelihoods of citizens. The purpose of this study is to explore possible scenarios for decreasing its incidence by combining population‐wide testing and/or social distancing policy.

## 2 MATERIALS AND METHODS

### 2.1 Model

**FIGURE 1:**
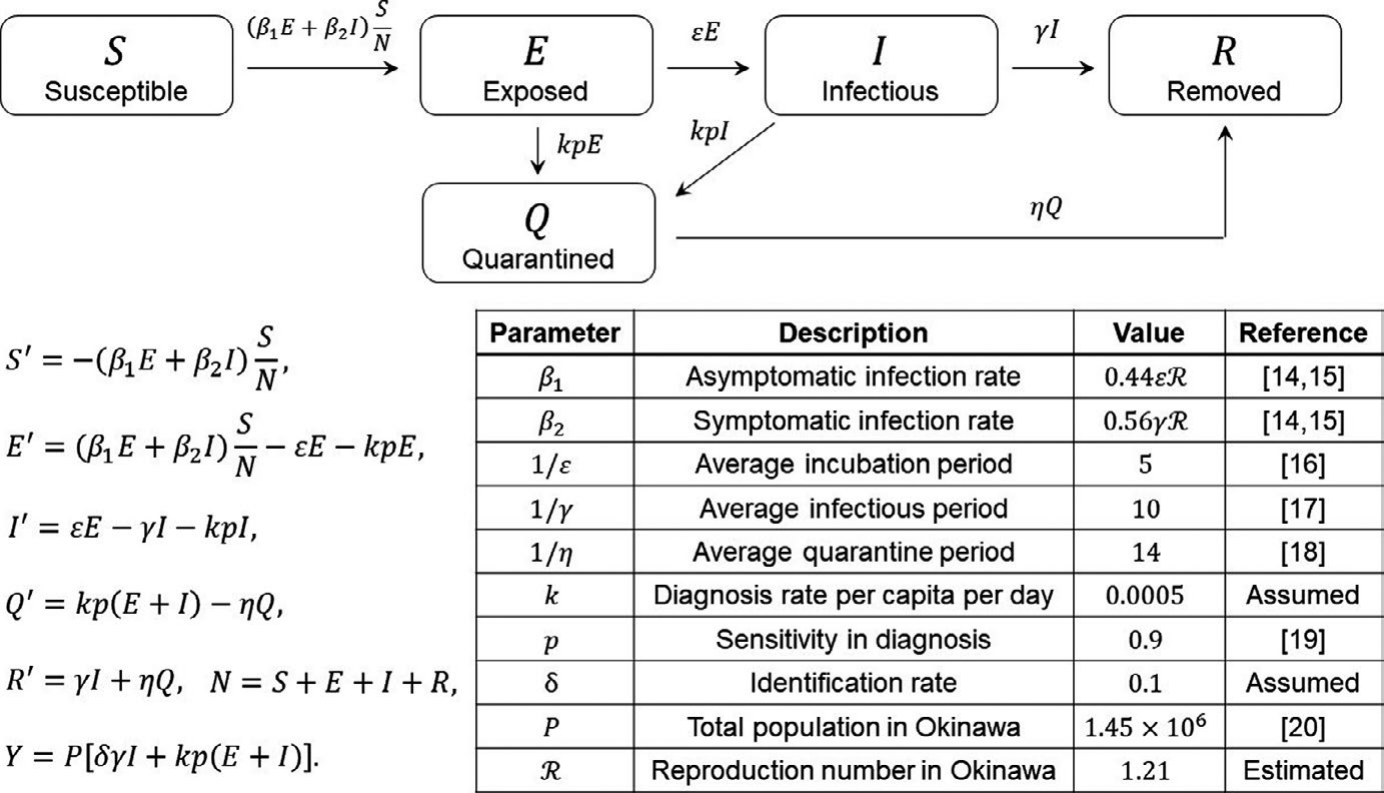
Transfer diagram, equations, and parameter table for our basic SEIQR model

Our basic model, an SEIQR compartmental model, is shown in Figure 1, in which the susceptible (S), exposed (asymptomatic infectious; (E), infectious (I), quarantined (Q), and removed (R) populations are considered.

In this model, *β*_1_ and *β*_2_ imply the asymptomatic and symptomatic infection rates, respectively.^14^, ^15^ *ε*, *γ*, and *η* imply the transition rates from E to I, I to R, and Q to R, respectively. That is, 1/*ε*, 1/*γ*, and 1/*η* imply the average periods of incubation, infection, and quarantine, respectively. We assumed that the unit time is 1 day and 1/*ε* = 5 (days), 1/*γ* = 10 (days), and 1/*η* = 14 (days) as shown in Figure 1, ^16^, ^17^, ^18^. As in Ref.,^14^ we assumed that the reproduction numbers for asymptomatic and symptomatic infections are *R*_1_ = 0.44*R* and *R*_2_ = 0.56*R*, respectively,^15^ where *R* denotes the reproduction number for COVID‐19 in Okinawa, and *β*_1_ and *β*_2_ are given by *β*_1_ = *εR*_1_ = 0.44*εR* and *β*_2_ = *γR*_2_ = 0.56*γR*, respectively. *k* is the diagnosis rate per capita per day in the whole population of Okinawa. We assumed that 0.05% (*k* = 0.0005) of the whole population of Okinawa can be diagnosed per day and only the exposed (E) and infectious (I) individuals can be detected with sensitivity *P* = 0.9,^19^ and transferred to the quarantined (Q) class. δ is the identification rate of infectious individuals. We assumed that the infectious individuals will be removed after the identification, and hence, (1 − δ)*γI* implies the density of newly removed individuals without identification. We chose the initial condition as S + E + I + Q + R = 1 so that each population implies the proportion to the total population. As shown in Figure 1, we can then regard *Y* = *P*[*δγ*I + kp(E + I)] as the number of newly reported cases, where *P* = 1.45 × 10^6^ denotes the total population in Okinawa.^20^ By fitting *Y* to the actual data of COVID‐19 in Okinawa from October 1 to November 30, 2020, by the least square method applied elsewhere,^21^, ^22^ we estimated the reproduction number *R* for Okinawa in this term as 1.21. The estimated epidemic curve and validation of the estimation can be seen in the top‐left of Figures, in which the blue curve represents the estimated epidemic curve, the red dots represent the data from October 1 to November 30, 2020, which was used for the estimation, and the green dots represent the actual data from December 1, 2020, to January 31, 2021.

### 2.2 Multiple scenarios

We simulated multiple scenarios under the assumption that the pulsed mass testing, a public health screening to recognize infected individuals, and social distancing policy for decreasing social contacts started on December 8, 2020. On the mass testing, we assumed that the testing is periodically carried out per 7/14/30 days to 5/25/50% of the susceptible and exposed populations in Okinawa. On the other hand, we also simulated a case that the daily self‐testing is carried out on weekdays to 1/5/10% of the susceptible and exposed populations in Okinawa. For simplicity, in the mass testing, we assumed a perfect test sensitivity for detecting asymptomatic infectious individuals^9^, regardless of specificity and compliance with quarantine. We obtained four scenarios  for the following cases.

1. Mass testing with testing rate 5% per 7/14/30 days;
2. Mass testing with testing rate 25% per 7/14/30 days;
3. Mass testing with testing rate 50% per 7/14/30 days;
4. Daily self‐testing with testing rate 1/5/10%.

In addition, in each scenario, we considered three cases of social distancing policies that decreased social contacts by 50%, and their duration was classified into three patterns: no lockdown, circuit breaker where the lockdown ends after 4 weeks, and the permanent lockdown.

## 3 RESULTS

**FIGURE 2:**
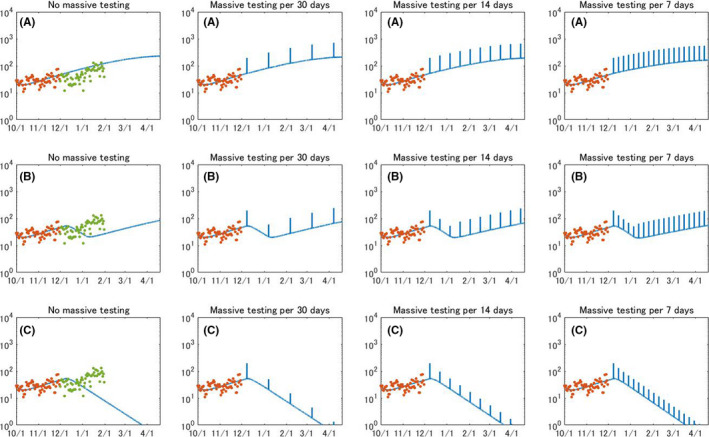
Evolution of daily reported COVID‐19 cases when population‐wide testing is performed for 5% of all citizens at specific intervals. (A) No lockdown, (B) circuit breaker, and (C) permanent lockdown

**FIGURE 3:**
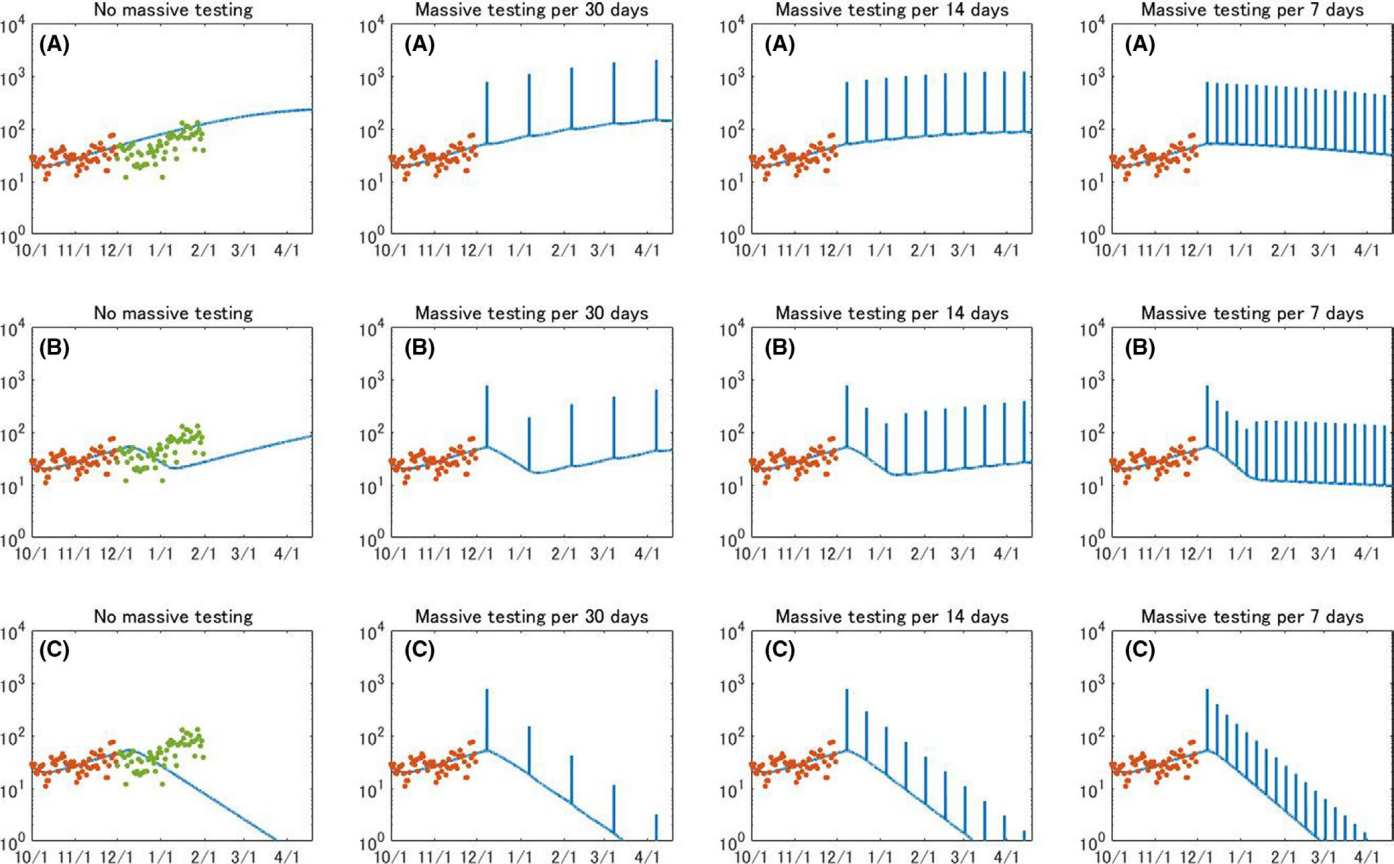
Evolution of daily reported COVID‐19 cases when population‐wide testing is performed for 25% of all citizens at specific intervals. (A) No lockdown, (B) circuit breaker, and (C) permanent lockdown

**FIGURE 4:**
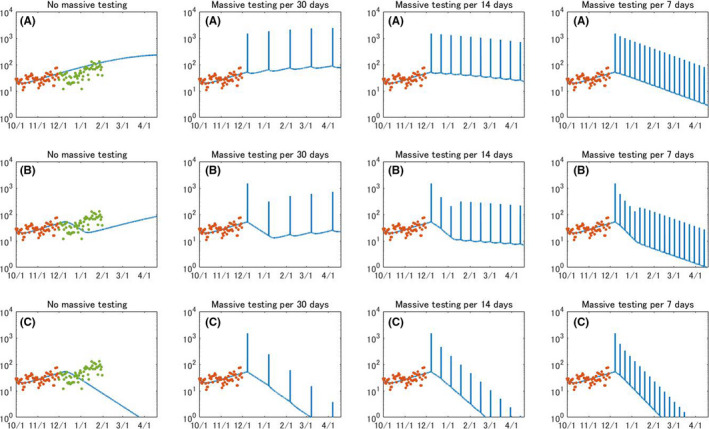
Evolution of daily reported COVID‐19 cases when population‐wide testing is performed for 50% of all citizens at specific intervals. (A) No lockdown, (B) circuit breaker, and (C) permanent lockdown

Multiple scenarios of mass testing toward 5%, 25%, and 50% of whole population in Okinawa at different intervals are presented in Figures 2, 3, 4. The number of daily reported cases will exponentially increase without mass testing or lockdown. While imposing the circuit breaker can curb the epidemic, it is not sufficient to aggressively suppress the virus; therefore, to decrease the number of patients, mass testing at appropriate intervals must be combined. The permanent lockdown will decrease COVID‐19 cases, and its period will be shortened by simultaneously implementing mass testing. Figure 2 clearly exhibits that mass testing for 5% of all citizens in Okinawa is not sufficient to curb an increasing number of daily reported cases, even when mass testing is conducted on a weekly basis or combined with circuit breaker.

This tendency slightly changes when 25% of whole population in Okinawa are screened, as presented in Figure 3. When the lockdown is not a policy option, mass testing per 7 days will alleviate an increasing trend; however, each mass testing continuously reports more than 100 cases for several months. When implementing mass testing per 7 days, an increasing trend will be reversed; however, it must be debatable in terms of health system capacity. Obviously, a frequent testing combined with social distancing policy will hugely contribute to breaking chains of transmission and vividly decreasing the reported number of COVID‐19 per mass testing. When combined with the circuit breaker, the daily reported number of cases will be decreased to below 20 in early January.

Figure 4 illustrates scenarios of mass testing that targets 50% of all citizens. Monthly mass testing will alleviate an increasing trend, but daily reported number of cases will amount to around 100 in April. Mass testing per 14 days will truly curb the epidemic, and the decreasing trend will be accelerated by simultaneously imposing a circuit breaker. When the weekly mass testing is performed without lockdown, it will promptly impact on crashing transmission dynamics and record less than 10 cases in late February. This period can be shortened to early January when combined with circuit breaker. Figure 4A,C also suggests that weekly mass testing for 50% of entire population will not only overturn an increasing trend of COVID‐19 patients but also bring a continuous decrease of patients, which is gained by simply imposing a permanent lockdown.

**FIGURE 5:**
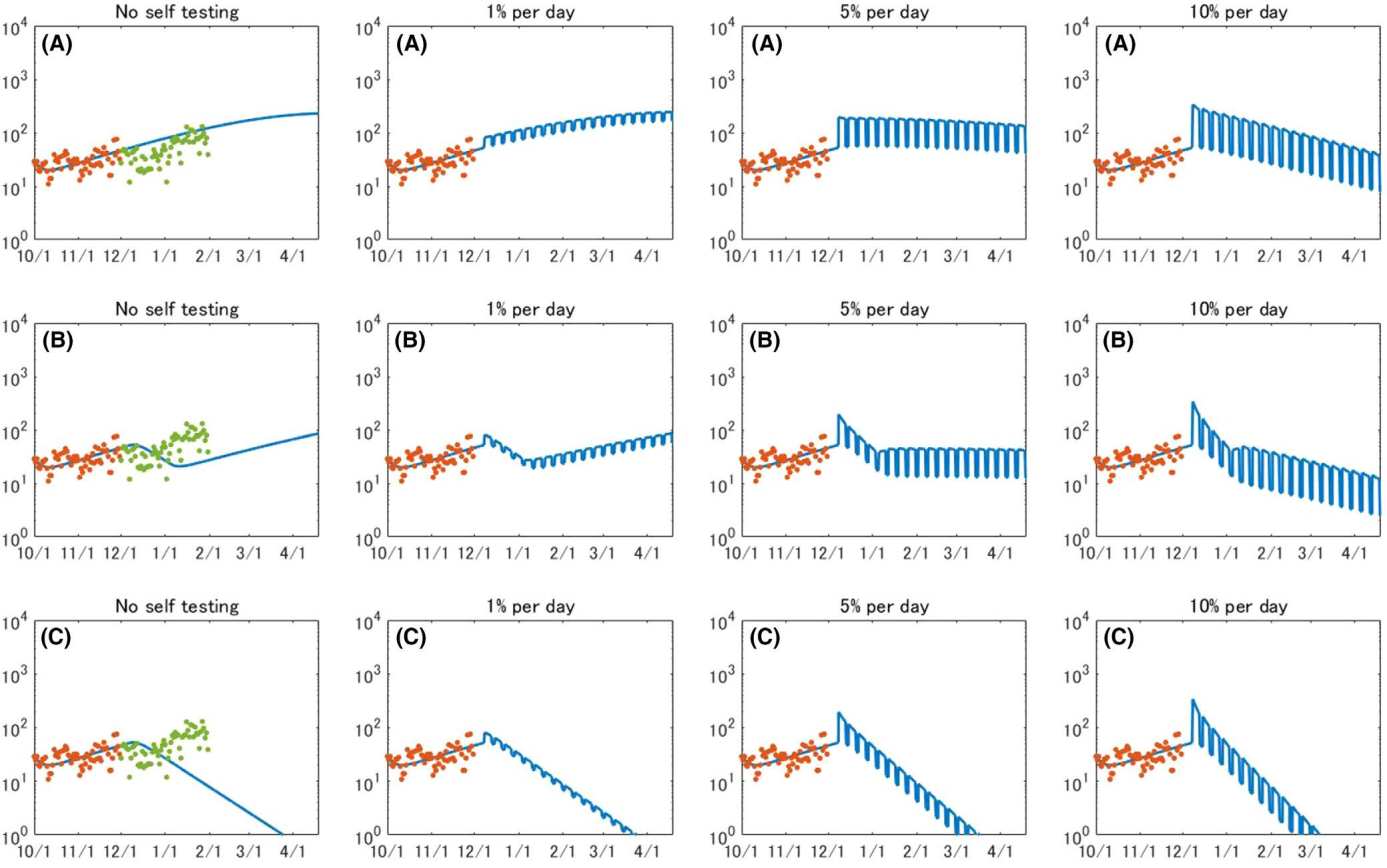
Evolution of daily reported COVID‐19 cases when population‐wide testing is performed on a daily basis. (A) No lockdown, (B) circuit breaker, and (C) permanent lockdown

Figure 5 presents the evolution of daily reported cases when the testing is conducted on a daily basis. Our results show that daily testing toward 1% of all citizens is not sufficient to hammer an increasing trend of daily reported cases without permanent lockdown. When 5% of all citizens are targeted, the number of reported cases will gradually decrease but it will record over 100 cases for several months. When 10% of all citizens were tested on a daily basis, the number of daily reported cases will reach below 100 in late February. When combined with circuit breaker, daily testing toward 1% of all citizens is not enough to turn the tide, but expanding more than 5% of all citizens will be effective to decrease the number of daily reported cases.

## 4 DISCUSSION

This study employed an SEIQR compartmental model to incorporate an impact of testing, isolation, and quarantine, which are primary pillars for source control management and critical to contain infectious diseases. Our modeling exercise briefly revealed three major findings. First, there were scenarios in Okinawa that regular population‐wide testing could curb an increasing trend of COVID‐19 patients and permanent lockdown could be averted, when properly organized and managed. As shown in green dots, maintaining the current test‐trace‐isolate (TTI) schemes was not enough to contain the deteriorating epidemiological situation of COVID‐19 pandemic, and thus, mobilizing sufficient target population for mass testing would be vital. Monthly screening would not bring a decrease in COVID‐19 infections, and ensuring adequate frequencies would be necessary. Second, mass testing presented implications for minimizing a period of lockdown. Third, to truly reverse an increasing trend of COVID‐19, circuit breaker, namely a short‐term lockdown, must be combined with population‐wide screening for sufficient population. Solely imposing a circuit breaker and slacking investment in TTI systems would not bring favorable outcomes in the long term.

Our results are in line with other several evidence. As presymptomatic and asymptomatic infections heavily contribute to the whole transmission of SARS‐CoV‐2,^23^ intervening these infections through regular essential medical services (ie, testing for symptomatic patients) is challenging. Considering the natural history of SARS‐CoV‐2 infection^24^ and high transmissibility from asymptomatic or presymptomatic infections,^15^, ^25^ capturing these infected individuals is a critical topic. In fact, some universities in the United States have already started to implement a frequent, intensive screening test by RT‐PCR.^26^, ^27^, ^28^ In addition, the necessity of frequent use of rapid, inexpensive tests, which can be positive for patients with infectiousness, has been critically argued in the United States,^29^, ^30^ and modeling studies support this presumption.^31^, ^32^ While Japan urgently needs to expand the testing capacity of RT‐qPCR for diagnosis, surveillance, and screening, using appropriate rapid testing kits for detecting highly transmissible patients can be considered when the testing capacity is overwhelmed. Although debatable, utilizing rapid testing tools for entry screening toward several high‐risk environments, such as full‐service restaurants and gathering events, may help prevent superspreading events. Moreover, our results indicate that implementing a population‐wide screening during a period of social distancing policy will accelerate a decreasing trend of SARS‐CoV‐2 infection. While this study assumed that only people tested positive would be isolated because the tracing capacity in Japan has already been overwhelmed and technical issues in contact tracing app. has been reported, utilizing a population‐wide screening as an opportunity for mass isolation and quarantine will aggressively reduce the transmission. In fact, a recent case study from the Slovak Republic^9^ suggested that population‐wide screening, even conducted only a few times, was beneficial for detecting COVID‐19 patients, appropriately isolating people who were tested positive, quarantining their close contacts, and breaking chains of transmission. If managed appropriately, this approach can contribute to minimizing the period of lockdown, thus mitigating the impact of social distancing policy on socioeconomic activities.

The COVID‐19 pandemic explicitly suggests that health is a foundation of society.^33^ As investment in public health, especially ramping up testing, will strengthen national health security and contribute to raising preparedness for other health emergencies in the near future, and a lower quality of life and death because of COVID‐19 are measured as economic loss,^34^ to what extent investing in public health infrastructure will positively work in containing the pandemic and revitalizing socioeconomic activities should be discussed from interdisciplinary perspectives. It should be also noted that fear for COVID‐19 among citizens will work as a big threat to the economy compared with several public health and social measures for preventing the spread of infection.^35^, ^36^ In fact, Okinawa faced a falling demand of 186.7 billion yen after the first wave of COVID‐19^37^ and that finally amounted at a scale of 648.2 billion yen in 2020.^38^ In entire Japan, the economy suffered from over 30 trillion yen.^39^ On the contrary, economies of scale have been positively working for reducing the price of PCR testing. Healthy Davis Together project in California, the United States, achieved a $6 for single test,^40^ and similar tendencies have been recognized in Japan. Introduction of pooling strategy, which has already been introduced in other countries,^41^, ^42^, ^43^ was finally approved in Japan in early 2021. It will further decrease the cost of testing. Generally, investment in public health will bring economic gain at several scales,^34^ because investment in public health will cost on millions of dollars but economic loss because of failure in containment will bring an economic loss at a scale of trillions of dollars.^34^ Advantages of minimizing economic loss by elimination strategy compared with suppression strategy have already been modeled,^44^ and a call for COVID‐19 elimination in Japan has been openly discussed.^13^ These windows of opportunities should be fully utilized. At the same time, several operational challenges must be noted. First, incentivizing public participation will be exigent. Discrimination against COVID‐19 patients; stigmatization toward workers in specific industries, such as health care, nightlife, and catering^13^, ^45^; and insufficient capacity in health communication will be eminent barriers for citizens to understand why mass testing contributes to protect the health of individuals and others. Second, a limited testing capacity and weak logistics in procuring personal protective equipment will be demanding. As public health centers in Japan have already been overwhelmed due to COVID‐19 pandemic, outsourcing its role for testing, fully utilizing academic laboratories, and incorporating testing capacities in private sectors will be vital. Third, the feasibility of mass screening schemes is completely different by context.^46^ A higher sensitivity for screening test is warranted, but using appropriate testing kits for truly recognizing infectiousness based on the previous discussion^47^ can be an option, especially when several challenges in gold standard testing, such as limited capacities of nucleic acid amplification test and prolonged turnaround times of testing results, remain unsolved. However, how to measure the infectiousness is a challenging question, as there is no clear threshold in defining infectiousness which presents a continuous trend. While lower values of cycle threshold seem to link with detecting culturable virus and higher chances of generating secondary cases,^48^, ^49^, ^50^, ^51^ careful interpretations of oversimplified modeling assumptions are warranted. Scant independent evaluations from real‐world data, several case reports that present lower sensitivity especially among asymptomatic cases, questionable workability of home‐based approaches because of differences in sampling methods, procurement and deployment of these tools, logistics for registering the data, and negative impacts of rapid testing results on modifying people's behaviors including false sense of security among citizens, breaking chains of COVID‐19, and protecting asymptomatic and presymptomatic COVID‐19 patients before their symptoms of onset will be eminent challenges.^52^, ^53^, ^54^, ^55^, ^56^ Discussing the feasibility in each context, summarizing advantages and disadvantages of testing tools for public health purposes, and intervals of mass testing will be vital research topics during COVID‐19 pandemic. While the false‐positive result was not a critical matter in population‐wide screening in the Slovak Republic,^9^ whether the population‐wide screening should use PCR testing or combined with rapid tests will be different between contexts and should be openly discussed. Fourth, our results suggest that mass testing will detect a number of patients. Therefore, there is an immense necessity of procuring isolation facilities. Launching shelter hospitals should be considered. These will not only work for truly breaking chains of transmission and avoiding additional transmissions (eg, household transmission), but lessen additional burden on tertiary referral hospitals. Fifth, social support, including financial scheme for positive cases, must be ensured with a political will to contain the pandemic. This is crucial to truly achieve a high compliance of isolation. Expanding the public support toward close contacts of patients who need to be quarantined will improve their compliance.

There are several limitations in this modeling exercise. First, the heterogeneity of COVID‐19 infection is not considered. The heterogeneity of the offspring distribution of secondary infection of COVID‐19 was recognized,^57^, ^58^ and intervention in high‐risk environments will be effective for preventing superspreading events.^59^ We launched this modeling exercise with concerns that extensive testing for containment has been rejected by scientific advisors,^13^ and a great number of essential workers, even healthcare workers at hospitals and nursing homes, still cannot access to regular protective screening in Japan.^12^ A random population‐wide testing was assumed on this modeling exercise, but opportunities for population‐wide testing will be beneficial for containing COVID‐19 and ensuring access to testing among citizens. Strategically mobilizing symptomatic patients and presymptomatic patients with minor symptoms of COVID‐19 for this opportunity will accelerate for advancing to containment. Second, the management of prefectural borders is not considered. However, as Okinawa prefecture has no land bridge except the US Military Bases and Facilities on Okinawa Island and Its Vicinity, test‐based strategy at its prefectural border can be easily implemented compared with other prefectures. Third, our modeling did not strongly address the importance of contact tracing. While this assumption is plausible in that the tracing capacity in public health centers was sometimes overwhelmed in Japan,^11^, ^60^ population‐wide testing followed by contact tracing activities, even limited ones, will contribute to further decreasing the transmission. Utilizing population‐wide screening as an opportunity for mass isolation can be a political option. Fourth, we assumed that the lockdown would decrease social contacts by 50%. Considering that the state of emergency in Japan in April has been declared under the assumption that social contacts will be decreased between 70% and 80%,^11^ our assumptions can be strengthened. Finally and importantly, securing sufficient resources will be discussed as major challenges. In those circumstances, promptly imposing a social distancing policy and employing targeted approaches based on the surveillance data will be alternative options. For example, results of wastewater surveillance for COVID‐19 need to be fully incorporated for containment efforts.

Despite these limitations, our modeling exercise presented several implications for the deteriorating situation of COVID‐19 in Okinawa, Japan. To end this pandemic, revive socioeconomic activities and protect citizens' lives and livelihoods, rigorous investment in public health is manifestly vital.^34^, ^61^ Testing obviously plays a vital role in containing the virus, and aggressive, widespread testing can capture more asymptomatic cases. Actually, countries advanced for near‐elimination conducted more testing per case.^62^ Population‐wide screening has been previously practiced for early containment or elimination,^7^, ^8^ and some countries in Europe started to employ this approach with their own social distancing policies. Our exercise implies that repeated mass testing that targets sufficient population will be a policy option to avoid the lockdown. However, it is easily expected that there are several challenges for promptly conducting a mass testing; in those circumstances, early introduction of a tight circuit breaker for saving time to build solid foundation of logistics for mass screening will be critical. In addition to expanding the testing capacity and ensuring citizens' access to testing, critical roles of extensive screening must be reconsidered for promptly detecting and protecting COVID‐19 patients, breaking further transmission dynamics, and containing the pandemic.

## CONFLICT OF INTEREST

The other authors have stated explicitly that there are no conflicts of interest in connection with this article.
